# Purification of Antioxidant Peptides by High Resolution Mass Spectrometry from Simulated Gastrointestinal Digestion Hydrolysates of Alaska Pollock (*Theragra chalcogramma*) Skin Collagen

**DOI:** 10.3390/md14100186

**Published:** 2016-10-17

**Authors:** Liping Sun, Weidan Chang, Qingyu Ma, Yongliang Zhuang

**Affiliations:** Yunnan Institute of Food Safety, Kunming University of Science and Technology, No. 727 South Jingming Road, Kunming 650500, Yunnan, China; kmlpsun@163.com (L.S.); changweidan08023@163.com (W.C.); maqingyu0323@163.com (Q.M.)

**Keywords:** Alaska pollock skin collagen, simulated gastrointestinal digestions, antioxidant peptide, peptide purification, de novo software, UniProt of MaxQuant

## Abstract

In this study, the stable collagen hydrolysate was prepared by alcalase hydrolysis and twice simulated gastrointestinal digestion from Alaska pollock skin. The characteristics of hydrolysates and antioxidant activities in vitro, including 2,2′-azino-bis (3-ethylbenzothiazoline-6-sulfonic acid) radical (ABTS^•+^) scavenging activity, ferric-reducing antioxidant power (FRAP) and hydroxyl radical (OH·) scavenging activity, were determined. After twice simulated gastrointestinal digestion of skin collagen (SGI-2), the degree of hydrolysis (DH) reached 26.17%. The main molecular weight fractions of SGI-2 were 1026.26 and 640.53 Da, accounting for 59.49% and 18.34%, respectively. Amino acid composition analysis showed that SGI-2 had high content of total hydrophobic amino acid (307.98/1000). With the simulated gastrointestinal digestion progressing, the antioxidant activities increased significantly (*p* < 0.05). SGI-2 was further purified by gel filtration chromatography, ion exchange chromatography and high performance liquid chromatography, and the A_1a3c–p_ fraction with high hydroxyl radical scavenging activity (IC50 = 7.63 μg/mL) was obtained. The molecular weights and amino acid sequences of key peptides of A_1a3c–p_ were analyzed using high resolution mass spectrometry (LC-ESI-LTQ-Orbitrap-MS) combined with de novo software and UniProt of MaxQuant software. Four peptides were identified from A_1a3c–p_, including YGCC (444.1137 Da) and DSSCSG (554.1642 Da) identified by de novo software and NNAEYYK (900.3978 Da) and PAGNVR (612.3344 Da) identified by UniProt of MaxQuant software. The molecular weights and amino acid sequences of four peptides were in accordance with the features of antioxidant peptides. The results indicated that different peptides were identified by different data analysis software according to spectrometry mass data. Considering the complexity of LC-ESI-LTQ-Orbitrap-MS, it was necessary to use the different methods to identify the key peptides from protein hydrolysates.

## 1. Introduction

Reactive oxygen species (ROS) and free radicals are very unstable and react rapidly with lipids and proteins in the body, generating damage in the biological system, such as DNA, protein and membrane lipid, if the human body cannot control their formation or eliminate them [1]. Therefore, it is important to inhibit oxidation reactions and the formation of free radicals in the living body. In recent years, some peptides have been found to possess antioxidant activity [2,3]. Collagen is rich in hydrophobic amino acids, and the abundance of these amino acids favors higher affinity to oil and better emulsifying ability. Therefore, collagen is expected to provide natural antioxidant peptides and exert higher antioxidant effects than other proteins. In addition, collagen peptides have good biocompatibility, good penetrability and cause no irritation to the body [4]. Researchers have reported the characteristic and antioxidant activity of different collagen resources, especially from aquatic animals, such as sea jumbo squid skin [5], jellyfish umbrella [2], smooth hound protein [6], sea cucumber [7], pacific hake [8] and tilapia skin [3].

The structure of bioactive peptides may be changed when they are digested, absorbed and transferred in gastrointestinal tract [9]. It is expected that bioactive peptides would not be further digested in the gastrointestinal tract and thereby ensure its stability after digestion. The stable peptides should ideally be isolated and identified in vivo. However, in vivo studies are costly and time-consuming, are rather complicated to perform. As an alternative to in vivo studies, a simple, rapid, and inexpensive in vitro simulated gastric and intestinal digestive method has been established to isolate bioactive peptides. In this method, the prior hydrolysis with the endopeptidases would increase the stability and bioavailability of the bioactive peptide in vivo. In the previous studies, it has been shown that gastrointestinal enzyme digestion results in more potent peptides compared with other enzymatic digestions [10].

Alaska pollock (*Theragra chalcogramma*) is one of the commercially important fish species in China, but a large number of scraps containing fish skin, head and bones are left in the processing of fillet production. It has been reported that 70% of the dry matter of fish skin is collagen. Some studies reported the characteristics of collagen and bioactivities of collagen peptides from Alaska pollock skin. Yan et al. [11] reported the characterization of collagen from the skin of Alaska pollock. Hou et al. [12] studied the immunomodulatory activity of Alaska pollock hydrolysates and the active peptide was identified. Byun and Kim [13] purified the key peptide from Alaska Pollock skin with angiotensin I converting enzyme inhibitory activity. Jia et al. [14] studied the enzymatic hydrolysates of Alaska Pollock skin with antioxidant activity.

In this study, twice simulated gastrointestinal (GI) digestion was used to prepare hydrolysates from the collagen of Alaska Pollock skin, in order to obtain the stable antioxidant peptides. The antioxidant activity of the hydrolysates was evaluated in vitro. The bioactive fraction from hydrolysates with the highest antioxidant activity was separated by gel filtration chromatography, ion exchange chromatography and high performance liquid chromatography. Furthermore, two methods, including de novo analysis software and UniProt of MaxQuant software, were used to identify the key peptides from protein hydrolysates.

## 2. Results and Discussion

### 2.1. Analyses, DH, Molecular Weight (MW) and Amino Acid Composition of Hydrolysates

**Figure 1:**
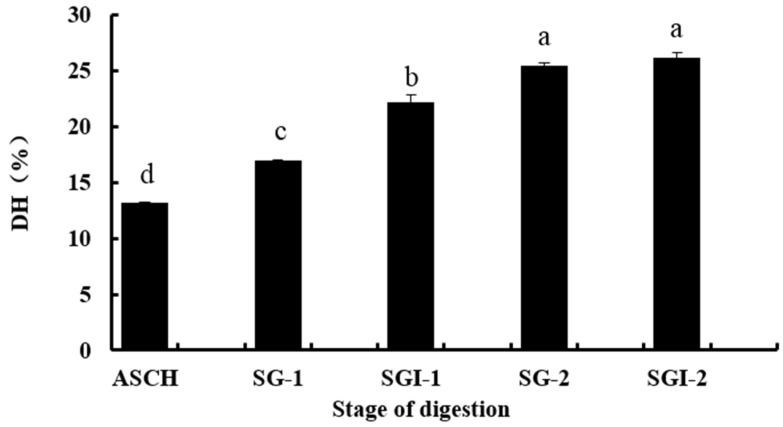
The hydrolysis degree changes of different stages of digestion from skin collagen of Alaska pollock ASCH: alcalase hydrolysates; SG-1: the first simulated gastric digestion; SGI-1: the first simulated intestinal digestion; SG-2: the second simulated gastric digestion; SGI-2: the second simulated intestinal digestion. Different letters indicated significant differences (*p* < 0.05).

In this study, Alaska Pollock skin collagen was hydrolysed by alcalase to obtain its hydrolysates (ASCH). Two successive simulated GI digestions of ASCH were conducted in order to get the stable collagen hydrolysates. The DHs of collagen hydrolysates were studied at different stages, including the first simulated gastric digestion (SG-1), the first simulated intestinal digestion (SGI-1), the second simulated gastric digestion (SG-2) and the second simulated intestinal digestion (SGI-2). As shown in Figure 1, the DH of ASCH was 13.17%. It increased from 16.92% (SG-1) to 22.65% (SGI-1) at the first simulated GI digestion. It was similar to the study of You et al. which indicated that more peptide bonds were broken using pancreatin digestion than using pepsin digestion [15]. In the second simulated GI digestion, the increase of DH was significant (*p* < 0.05) at the stage of SG-2 (25.47%), but it was not significant between the SG-2 and SGI-2 stage (26.17%) (*p* > 0.05). It might be because the DH became basically stable as the second simulated intestinal digestion was processed.

Furthermore, the molecular weight distributions of the different stages of digestion from skin collagen of Alaska pollock were shown in Table 1. The main molecular weight fractions of ASCH, SGI-1 and SGI-2 were 3198.76 (66.82%), 1552.34 (74.66%) and 1026.26 Da (59.49%), respectively. With the increasing of DH, the molecular weight of the hydrolysates significantly decreased. As shown in Table 1, the MW distribution of SGI-2 was 1026.26, 640.53, 284.97 and 96.58 Da. Based on the peak area, they accounted for 59.49, 18.34, 16.60 and 4.56%, respectively.

The amino acid compositions of the different stages of digestion were showed in Table 2. The compositions of three stages were similar and they were rich in Gly, Glu, Pro, Asp, and Arg. The result was similar to that of other fish skin hydrolysates [4,5] and in accordance with the characteristics of collagen hydrolysates. In this study, the total hydrophobic amino acid (THAA) contents of three hydrolysates were high, showing 315.11, 302.22 and 307.98 per 1000 residues, respectively. High content of hydrophobic amino acids could increase the solubility in lipids and therefore enhance the antioxidative activity of hydrolysates [4].

### 2.2. Analyses of Antioxidant Activities

**Figure 2:**
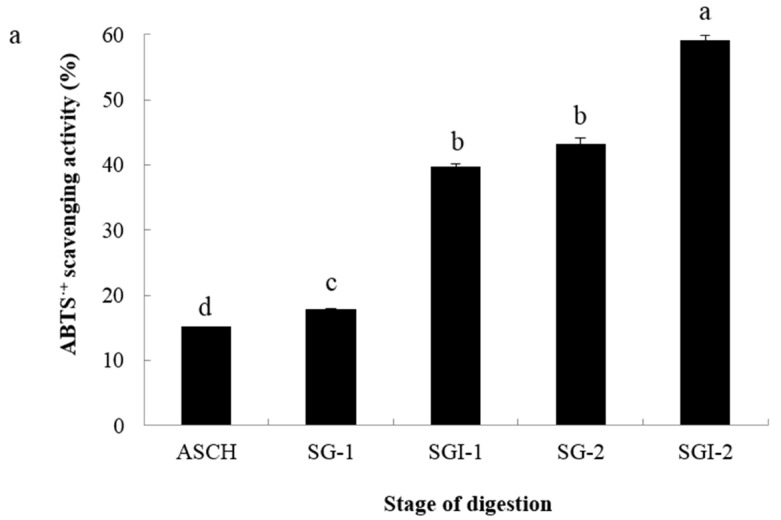

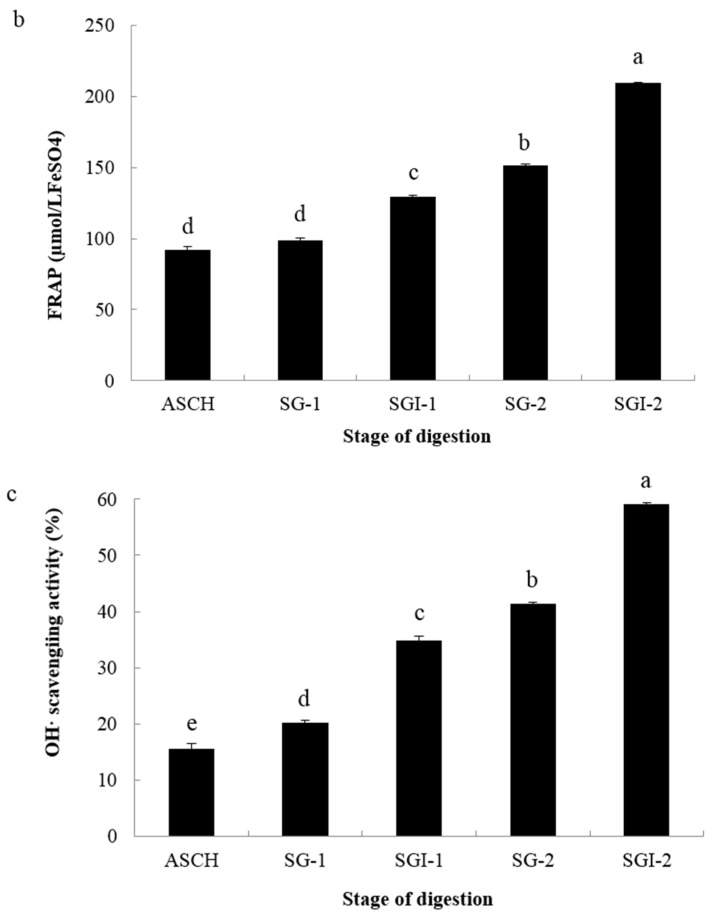
The antioxidant acitivties of different stages of digestion of skin collagen of Alaska pollock. (**a**): ABTS^•+^ scavenging activity (at 0.5 mg/mL); (**b**): FRAP (at 10 mg/mL); (**c**): OH· scavenging activity (at 2 mg/mL). Different letters indicated significant differences (*p* < 0.05).

Since there is no single antioxidant standard method to test for antioxidant capacity, it is recommended to use different methods for investigating the different mechanisms of antioxidant capacity [16]. In order to evaluate the antioxidant activity of collagen hydrolysates, the pHs was adjusted to about 7.0 in this study. As shown in Figure 2, the ABTS^•+^ scavenging activity, FRAP and OH· scavenging activity were measured.

The ABTS assay is often used to evaluate the ability of antioxidants to scavenge free radicals. After simulated GI digestion, the ABTS^•+^ scavenging activity obviously increased (Figure 2a). The ABTS^•+^ scavenging activity was 39.79% in the SGI-1 stage and 59.17% in the SGI-2 stage at the concentrations of 0.5 mg/mL. The FRAP assay is based on the ability of antioxidants to reduce Fe^3+^ to Fe^2+^ in the presence of 1,3,5-tri(2-pyridyl)-2,4,6-triazine (TPTZ). As shown in Figure 2b, with the simulated gastrointestinal digestion progressing, FRAP of the hydrolysates increased significantly (*p* < 0.05). At the dose of 10 mg/mL, the activities of SGI-1 and SGI-2 were 129.42 and 209.27 μmol/L FeSO_4_, respectively. Hydroxyl radical is the most reactive radical, which has been demonstrated to be a highly damaging species in free radical pathology, attacking almost every molecule in living cells. As shown in Figure 2c, the simulated GI digestion significantly increased the OH· scavenging activity (*p* < 0.05). Indeed, the OH· scavenging activity of ASCH was 15.53% at the concentration of 2 mg/mL, and the activities of SGI-1 and SGI-2 were 34.77 and 59.11%, respectively. The increase of scavenging activity of the pancreatin digestion process was higher than the pepsin digestion.

### 2.3. Purification of the Antioxidant Peptides from SGI-2

The removal of OH· is probably one of the most effective defenses of a living body against various diseases [17]. Based on this reason, the OH· scavenging activity was selected as the indicator of purification of antioxidant peptides in the study.

**Figure 3:**
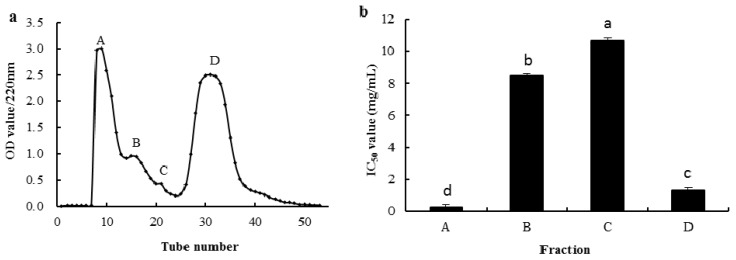
Sephadex G-25 gel chromatography (**a**) and the IC_50_ value (mg/mL) of each fraction was measured by OH· scavenging activities (**b**). Different letters indicate significant differences (*p* < 0.05).

The SGI-2 solution was purified by a Sephadex G-25 gel filtration column, and four fractions were obtained, noting A–D respectively (Figure 3a). Four fractions were collected, concentrated and the OH· scavenging activities were determined. Results showed that fraction A had the highest OH· scavenging activity among the four fractions, with the IC_50_ value being 0.26 mg/mL (Figure 3b).

**Figure 4:**
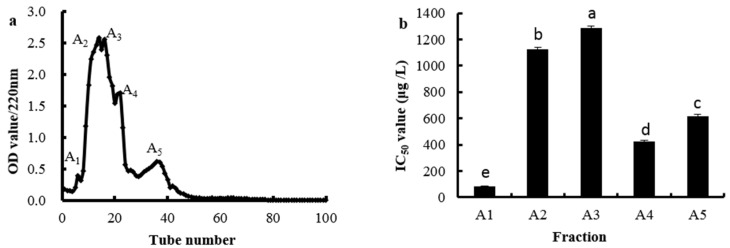
Elution profile of fraction A separated by SP Sephadex C-25 chromatography (**a**) and the IC_50_ value (μg/mL) of the OH· scavenging activities of each fraction (**b**). Different letters indicate significant differences (*p* < 0.05).

Ion-exchange chromatography was a method of separation according to the substance with a different acid-base property and polarity. SP Sephadex C-25 was one of the strong cation exchangers with a main functional group of sulfopropyl and it was widely used in separating bioactive peptides [18]. The fraction A collected from Sephadex G-25 was further separated by the SP Sephadex C-25 column and five fractions were obtained, noting A_1_, A_2_, A_3_, A_4_ and A_5_, respectively (Figure 4a). The OH· scavenging activities of these five fractions were shown in Figure 4b; fraction A_1_ had the highest OH· scavenging activity with the IC_50_ value being 81.15 μg/mL. Thus, the fraction A_1_ was selected for next separation.

**Figure 5:**
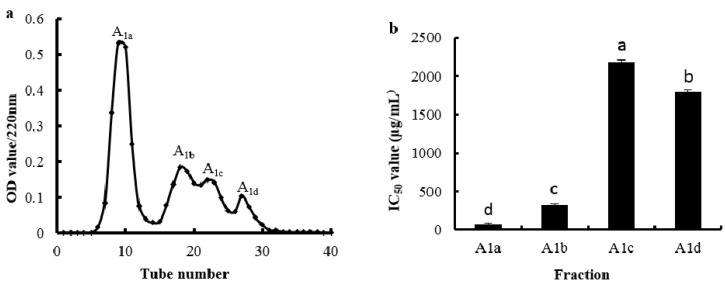
Elution profile of fraction A_1_ separated by Sephadex G-15 chromatography (**a**) and the IC_50_ value (μg/mL) of the OH· scavenging activities of each fraction (**b**). Different letters indicate significant differences (*p* < 0.05).

Sephadex G-15 was used to remove NaCl from the eluate of SP Sephadex C-25 in the fraction A_1_. As shown in Figure 5, four fractions (A_1a_, A_1b_, A_1c_, and A_1d_) were obtained and their OH· scavenging activities were measured. The OH· scavenging activity of the fraction A_1a_ was the highest compared with the other three fractions, and the IC_50_ value was 73.52 μg/mL.

**Figure 6:**
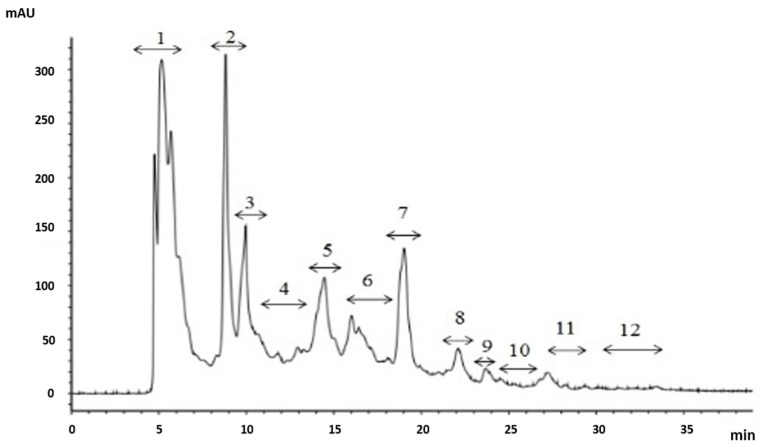
Chromatography of A_1a_ separated by a Shim-pack GIS C18. Liner gradient was 5%–30% acetonitrile containing 0.1% TFA from 0 to 30 min.

The fraction A_1a_ was further isolated by a Shim-pack GIS C18 preparative column with a liner gradient of acetonitrile containing 0.1% trifluoroacetic acid (TFA) from 5% to 30% in 30 min. The elution profile was shown in Figure 6. A total of 12 peaks were obtained and named as A_1a1_–A_1a12_, respectively. Each peak was collected, concentrated and it’s OH· scavenging activities were measured. The result showed that A_1a3_ had the highest antioxidant activity and the IC_50_ value of A_1a3_ was 31.72 μg/mL.

**Figure 7:**
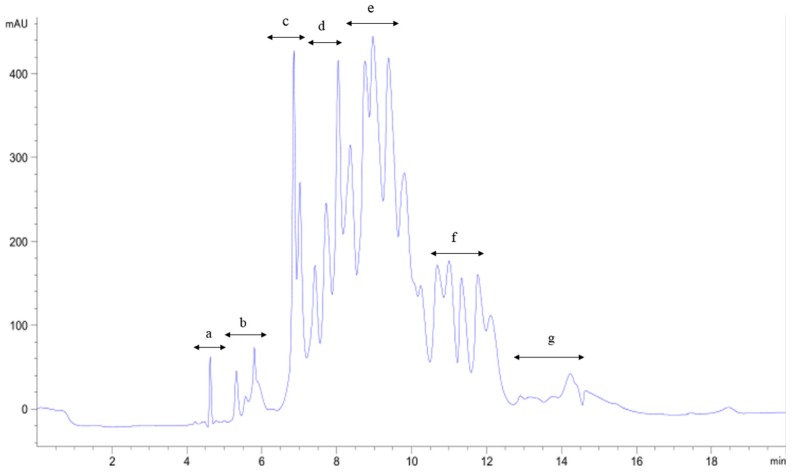
Chromatography of A_1a3_ separated by C18 semi-preparing HPLC. The liner gradient was 5%–25% acetonitrile containing 0.1% TFA from 0 to 30 min.

**Figure 8:**
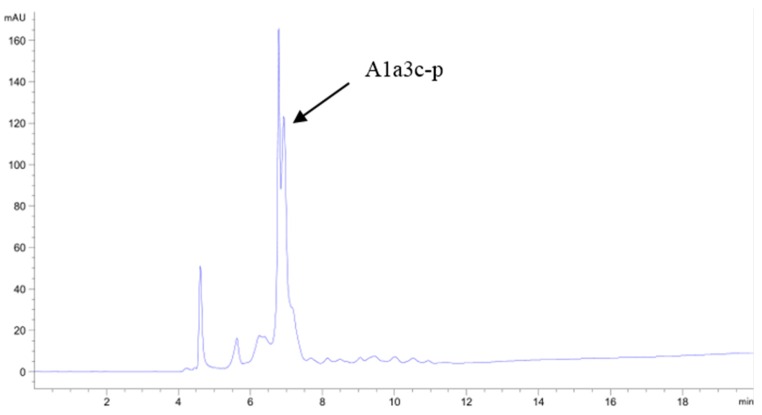
Chromatography of A_1a3c_ separated by C18 semi-preparing HPLC. The liner gradient was 5%–20% acetonitrile containing 0.1% TFA from 0 to 30 min.

The fraction A_1a3_ was isolated by HPLC on the semi-preparative C18 column using a liner gradient of acetonitrile containing 0.1% TFA from 5% to 25% in 30 min. Seven fractions were collected and designated as A_1a3a_–A_1a3g_ in turn respectively (Figure 7). After the OH· scavenging activities of seven fractions were determined, we found that A_1a3c_ had the highest OH· scavenging activity, and the IC_50_ value of A_1a3c_ was 14.36 μg/mL. The most active A_1a3c_ faction was isolated again by the semi-preparative C18 column using a different liner gradient of acetonitrile containing 0.1% TFA from 5% to 20% in 30 min. The main peak A_1a3c–p_ with high antioxidant activity was collected and concentrated (Figure 8). The IC_50_ value of OH· scavenging activity of the A_1a3c–p_ fraction was 7.63 μg/mL.

### 2.4. Identification of Purified Peptide

**Figure 9:**
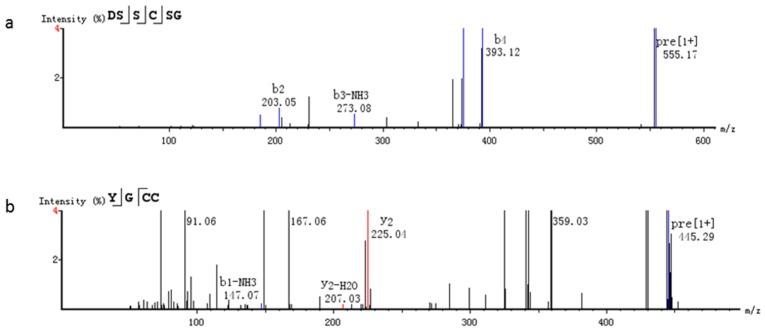
MS/MS spectrum analysis of the active peptides. (**a**): DSSCSG; (**b**): YGCC.

The antioxidant activity of peptides is connected with their molecular weights, amino acid compositions, amino acid sequences and so on [19]. In this study, the fraction A_1a3c–p_ was analyzed by high resolution mass spectrometry combined with two methods, including de novo software and MaxQuant software. As shown in Figure 9, two peptides were obtained by de novo software. The peptide sequences were Try–Gly–Cys–Cys (YGCC) and Asp–Ser–Ser–Cys–Ser–Gly (DSSCSG), and their molecular weight was 444.1137 and 554.1642 Da, respectively. The data was scanned in the “fish collagen” database by UniProt of MaxQuant software. Two peptides, Asn-Asn-Ala-Gln-Tyr-Tyr-Lys (NNAEYYK) and Pro-Ala-Gly-Asn-Val-Arg (PAGNVR), were identified, and their molecular weight was 900.3978 and 612.3344 Da, respectively. The amino acids at the *C*-terminus of two peptides were K and R, which conformed to the fracture characters of simulated gastric and intestinal digestions.

Generally, there is no direct relationship between antioxidant activity and molecular weight. However, the previous study indicated that the peptides with smaller molecular weights have stronger antioxidant activities, more resistant to the gastrointestinal digestion and easier to cross the intestinal barrier to exert biological activities than larger peptides [20]. Antioxidative peptides usually contain 2–20 amino acids with molecular weights below 3000 Da [5]. In this study, the amino acid numbers of four peptides identified from Alaska pollock skin collagen were 4, 6, 7, and 6 and the molecular weight was lower 1000 Da. The amino acid number and molecular weight was in accordance with the feature of antioxidant peptide. Our study was similar to the peptides purified from walnut [21] and loach protein hydrolysates [15].

Moreover, compositions and the specific position of amino acids in the peptide may play an important role in its antioxidant activities. High content of hydrophobic amino acids, especially at the *N*- or *C*-terminus of peptides, could enhance the activities of antioxidative peptides by interacting with lipid molecules and donating protons into radicals to scavenge radicals [22]. Moreover, polar/charged amino acids such as Arg at the *C*-terminus position also contribute to the antioxidant activity [23]. Our results were similar to these previous reports, and hydrophobic amino acids or arginine existed in the terminus of four peptides.

Some studies have reported that peptide sequences containing Tyr show strong antioxidant activity, especially when the presence of Tyr was at terminals of the peptide sequence. The antioxidant activity of Tyr may be explained by the special capability of phenolic groups to serve as hydrogen donors, which is one mechanism of inhibiting the radical-mediated peroxidizing chain reaction [24]. YGCC obtained from this study had a Tyrat *N*-terminus, and this might be one of the reasons why YGCC showed higher radical scavenging activity. In addition, NNAEYYK had two Tyr, which could effectively increase its antioxidant activity. Previous studies show that Cys is hydrophobic in nature and can interact directly with free radicals by donating the sulfur hydrogen, so the presence of Cys is one of the reasons for the good antioxidant activity of the isolated peptide [25]. Li et al. considered that Cys residue at the *C*-terminus or next to the *C*-terminus plays an important role in antioxidative activities [26]. It was similar to our study and YGCC and DSSCSG contained Cys, YGCC in particular had two Cys at the *C*-terminus, which might improve its antioxidant activity. Acidic amino acids, such as Asn and Gln, play important roles in the chelation of metal ions by their side chains, which may inhibit the formation of the hydroxyl radical [27]. Rajapakse et al. reported that the presence of Asp seemed to play a vital role, irrespective of its position, as observed in several antioxidative peptide sequences [28]. It was similar to our results. DSSCSG and NNAEYYK had Asp and Asn at the *N*-terminus, respectively.

The fraction A_1a3c–p_ was analyzed using high resolution mass spectrometry, and the key peptides obtained by de novo and MaxQuant software were different. Therefore, to adequately identify the key peptides from protein hydrolysates fractions, it was necessary to use different methods to analyze mass data. A further study about the quantitative analysis of key peptides will be carried out.

## 3. Materials and Methods

### 3.1. Materials

Collagen of Alaska pollock skin was prepared by the previous methods. Alcalase was purchase from Genencor International Co. (Wuxi, China); Pepsin, pancreatin, 2,2′-azino-bis (3-ethylbenzothiazoline-6-sulfonic acid) (ABTS) and 2,4,6-Tris(2-pyridyl)-*S*-triazine (TPTZ) were purchased from Sigma Chemical Co., (St. Louis, MO, USA). Sephadex G-25, Sephadex G-15 and SP Sephadex C-25 were purchased from GE Healthcare (Fairfield, CT, USA). Acetonitrile (HPLC grade) was purchased from Merck KGaA (Darmstadt, Germany). All other reagents used in this study were analytical grade.

### 3.2. Preparation of Skin Collagen Hydrolysates of Alaska Pollock

The Alaska pollock skin collagen was mixed with distilled water at a concentration of 1% (*w*/*v*). The mixture was adjusted to pH 9.0 by 1 M NaOH solution and then hydrolyzed using Alcalase (E/S: 5/100, *w*/*w*) at 55 °C for 2 h. Alcalase were inactivated in boiling water for 10 min and centrifuged at 5000 rpm for 20 min. The supernatants (ASCH) were collected and then lyophilized. ASCH was hydrolyzed by the simulated GI digestion [15,29]. A total of 2 g ASCH was dissolved in 150 mL distilled water and adjusted to pH 2.5 with 6 M HCl. Then, pepsin was added at a ratio of enzyme to substrate of 1:35 (*w*/*w*). After the mixture was incubated at 37 °C for 1 h with shaking (SG-1), sodium cholate (0.2 mM) and SG-1 (1:1, *v*/*v*) were mixed. The pH was adjusted to 7.5 using 2 M NaOH. Then, pancreatin was added at a ratio of enzyme to substrate of 1:25 (*w*/*w*). The mixture was incubated at 37 °C for 2 h with shaking and then inactivated in boiling water for 15 min and centrifuged at 5000 rpm for 20 min. The supernatants (SGI-1) were desalinized and then lyophilized. The second simulated gastric and intestinal digestions were conducted using the same method with the above processes and SG-2 and SGI-2 were obtained.

### 3.3. Determination of Characteristics of Hydrolysates

#### 3.3.1. Determination of the Degree of Hydrolysis (DH)

The contents of free amino (–NH_2_) and protein (*N*) were evaluated according to the ninhydrin colorimetric method and kjeldah method, respectively. DH was calculated as follows [30]:

(1) $$DH=\frac{h(mmol/g)}{h_{\mathrm{tot}}(mmol/g)}\times 100\%=[\frac{M_{1}(\mu mol/mL)}{N(mg/mL)}-M_{0}(mmol/g)]÷h_{\mathrm{tot}}(mmol/g)\times 100\%$$

where *h* is the number of broken peptide bonds per gram protein; *h*_tot_ is the total number of peptide bonds per gram original protein (the *h*_tot_ of collagen was 8.41 mmol per gram protein); *M*_1_ is the content of –NH_2_ in hydrolysate; *M*_0_ is the content of –NH_2_ in original protein; *N* is the content of protein in hydrolysate.

#### 3.3.2. Molecular Weight (MW) Distribution

The molecular weight distribution of the different hydrolysates was measured using a high-performance liquid chromatography (HPLC) system (1260 series, Agilent Scientific, Santa Clara, CA, USA) with a TSK gel 3000 PWXL column (30 mm i.d. × 7.8 mm, Tosoh, Tokyo, Japan) [17]. The mobile phases were acetonitrile-water (1:1, *v*/*v*) in the presence of 0.1% (*v*/*v*) trifluoroacetic acid, and the flow rate was 0.6 mL/min. The process was monitored at 220 nm at 30 °C. A calibration curve of molecular weight was prepared according to the following standards: cytochrome C (12,500 Da), insulin (5734 Da), vitamin B_12_ (1355 Da), hippuryl-histydilleucine (429.5 Da), and glutathione (309.5 Da). The logarithm of molecular weight (MW) and the retention time (*tR*) were in a linear relationship and the formula was calculated as lg MW = −0.284*tR* + 7.310 (*R*^2^ = 0.9922, *p* < 0.01).

#### 3.3.3. Amino Acid Composition

The different hydrolysates were hydrolyzed under reduced pressure with 6 mol/L HCl at 110 °C for 22 h and the amino acid compositions were analyzed on a Hitachi amino acid analyzer 835-50 (Hitachi, Tokyo, Japan).

### 3.4. Antioxidative Activity Assay

#### 3.4.1. ABTS^•+^ Scavenging Activity Assay

ABTS^•+^ scavenging activities were determined as described by previous method with a slight modification [31]. A total of 5 mL of 7 mM ABTS and 88 μL of 40 mM potassium persulfate was mixed to prepare ABTS^•+^ stock solution. The mixture was left in the dark at room temperature for 12 h. The ABTS^•+^ stock solution was diluted with PBS (2 mM, pH 7.4) to an absorbance of 0.70 ± 0.02 at 734 nm. Then, 0.5 mL of samples were mixed with 4 mL ABTS^•+^ stock solution. The mixture was shaken for 10 s and left in the 30 °C water bath for 6 min. The absorbance was measured at 734 nm. The capability of ABTS^•+^ scavenging was calculated according to the following equation.

(2) $$\mathrm{Radical}\;\mathrm{scavenging}\;\mathrm{activity}\;(\%)=\frac{A_{c}-(A_{s}-A_{cs})}{A_{c}}\times 100$$

where *A*_c_ was 0.5 mL ethanol + 4.0 mL ABTS^•+^ solution; *A*_s_ was 0.5 mL sample + 4.0 mL ABTS^•+^ solution; *A*_cs_ was 0.5 mL sample + 4.0 mL ethanol.

#### 3.4.2. FRAP Assay

FRAP was determined according to the method of Alemania et al. [32] with a slight modification. A total of 300 mM acetic acid buffer solution (pH 3.6) was mixed with 10 mM TPTZ and 20 mM FeCl_3_∙6H_2_O according to the rate of 10:1:1. Then the mixture was left in a 37 °C water bath to prepare the FRAP solution. The mixture of 150 μL samples and 4.5 mL FRAP solution was reacted at 37 °C for 10 min and then determined the absorbance at 593 nm. A total of 150 μL of distilled water was used instead of samples solution as a control. The absorbance of different concentrations of FeSO_4_ solution (0–500 μmol/mL) were determined at 593 nm. The FRAP of samples were expressed as equal to μmol/mL FeSO_4_.

#### 3.4.3. OH· Scavenging Activity Assay

Hydroxyl radical (OH·) scavenging activity was determined by the previous method with slight modification [33]. Briefly, after 1 mL of samples mixed with 0.3 mL of FeSO_4_ (8 mM), 1 mL of salicylic acid (3 mM) and 0.25 mL of H_2_O_2_ (20 mM), the mixture was incubated at 37 °C for 30 min. The reaction mixture was cooled by flowing water to room temperature. Then, 0.45 mL distilled water was added into the mixture to make the end volume 3.0 mL. The mixture was centrifuged at 3000 rpm for 10 min. The absorbance of supernatant was measured at 510 nm, and 1 mL of the solvent solution was used instead of the sample solution as a control. The capability of scavenging the hydroxyl radical was calculated according to following equation:

(3) $$\mathrm{Radical}\;\mathrm{scavenging}\;\mathrm{activity}\;(\%)=\frac{A_{0}-(A_{1}-A_{2})}{A_{0}}\times 100$$

where *A*_0_ was the absorbance of the control without a sample, *A*_1_ was the absorbance with a sample, and *A*_2_ was the absorbance of the reagent blank. The IC_50_ value was defined as an effective concentration that is required to scavenge 50% of radical activity.

### 3.5. Purification of Antioxidant Peptides

The SGI-2 was dissolved in distilled water and preliminarily separated by a Sephadex G-25 gel filtration column (Φ 2.6 cm × 30 cm). The SGI-2 was eluted at a flow rate of 0.5 mL/min and collected every 6 min. Then, the eluted solution was monitored at 220 nm. The peptide fraction showing the highest OH· scavenging activity was collected and concentrated.

The peptide fraction with the highest OH· scavenging activity was loaded onto a SP Sephadex C-25 of cationic exchange column (Φ 1.6 cm × 80 cm), which was previously equilibrated with a 0.02 M sodium acetate buffer (pH 4.0). The peptide fraction was eluted with a linear gradient of NaCl concentration from 0 to 1.0 M in the same buffer at a flow rate of 0.8 mL/min and monitored at 220 nm. The elution solution was collected at 6 min intervals and then concentrated. The OH· scavenging activity of isolated fractions was determined.

The peptide fraction with the highest OH· scavenging activity obtained from SP Sephadex C-25 was dissolved and further separated and desalinated by the Sephadex G-15 gel filtration column (Φ 2.6 cm × 30 cm). The peptide fraction was eluted at a flow rate of 0.5 mL/min and collected every 6 min. The solution was monitored at 220 nm. The OH· scavenging activity of isolated fractions was determined.

The highest active faction after Sephadex G-15 was further purified by preparative high performance liquid on a Shim-pack GIS C18 preparative column (Φ 20 mm × 250 mm, Shimadzu, Kyoto, Japan). The mobile phase A was water, and mobile phase B was acetonitrile containing 0.1% TFA. The column was eluted by a linear gradient of 5% B to 30% B in 30 min. The flow rate was 10.0 mL/min, and detection wavelength was 220 nm. The above steps were repeated several times until the different eluted fractions were able to measure the OH· scavenging activity and purify further. The same fractions were pooled and concentrated to remove acetonitrile and TFA.

The fraction with the highest OH· scavenging activity was passed through a Zorbax semi-preparative SB-C18 column (Φ 9.4 mm × 250 mm, Agilent Scientific, Santa Clara, CA, USA) by Agilent HPLC 1260 system (Agilent Scientific, Santa Clara, CA, USA). The fraction was eluted using a linear gradient of 5% to 25% acetonitrile containing 0.1% TFA (0 to 30 min) at a flow rate of 2.0 mL/min [3]. The column temperature was controlled at 35 °C and the detection wavelength was 220 nm. The fraction showing the high antioxidant activities was concentrated to remove acetonitrile and TFA and lyophilized.

### 3.6. Analysis and Identification of Purified Peptide

#### 3.6.1. Assay of High Resolution Mass Spectrometry (LC-ESI-LTQ-Orbitrap-MS)

Purified peptides were eluted from Q Exactive Focus (Thermo Fisher, Tewksbury, MA, USA) with a Hypersil Gold C18 chromatographic column (1.9 μm, Φ 2.1 mm × 100 mm) at a flow rate of 0.2 mL/min. The mobile phase A was acetonitrile containing 0.1% formic acid, and mobile phase B was water with 0.1% formic acid. The column was equilibrated for 1 min at 5% A and eluted as the following flow gradient: 1–2.5 min, 5.0%–10.0% A; 2.5–12.5 min, 10.0%–25.0% A; 12.5–20 min, 25.0%–52.5% A; 20–22 min, 52.5%–95.0% A; 22–24 min, 95.0%–5.0% A; 24–30 min, 5.0% A. The mass spectrogram was scanned in the positive ion mode. The instrument was set up as follows: scanning mode, Full MS-ddMS2; resolution, Full MS 35000, ddMS2 17500; scan range: 120~1800 *m*/*z*; stepped CE: 10 eV, 20 eV, 30 eV; AGC target: 1 × 10^5^.

#### 3.6.2. Identification of the Key Peptides

The molecular weights and amino acid sequences of purified peptides were identified by two software methods: (1) De novo analysis software. The peptide was automatically selected for fragmentation. The molecular weight and amino acid sequence of the MS date was processed using de novo software. Peptide identifications were accepted if they could be established at greater than 85% probability; (2) UniProt of MaxQuant software. Peptides identification was achieved by comparing mass data against the UniProt data using MaxQuant Server (version 1.5.3.28) [34]. The “fishcollagen” database was downloaded from http://www.uniprot.org/. The parameters of database searches were as follows: variable oxidation of methionins, and tolerance of the ions at 5 ppm for parents and 0.5 Da for fragments [35]. No enzyme or static modification was set for database searching. No missed cleavage was allowed.

### 3.7. Statistical Analysis

All results obtained were expressed as means ± standard deviation and analyzed by the SPSS 19.0 statistical software (Armonk, NY, USA). Data were analyzed using one-way analysis of variance (ANOVA). *p* < 0.05 indicated statistical significance.

## 4. Conclusions

In this study, the stable collagen hydrolysate of Alaska pollock skin was prepared by successive simulated gastrointestinal digestion. The DHs, molecular weight distributions, amino acid compositions and antioxidant activities in vitro were evaluated. With the simulated gastrointestinal digestions, The DHs and antioxidant activities increased obviously. An antioxidant fraction (A_1a3c–p_) was purified by gel filtration chromatography, ion exchange chromatography and high performance liquid chromatography, and the IC_50_ value of hydroxyl radical scavenging activity was 7.63 μg/mL. Furthermore, four key peptides of A_1a3c–p_, including YGCC, DSSCSG, NNAEYYK and PAGNVR, were analyzed by high resolution mass spectrometry combined with de novo software and UniProt of MaxQuant software. This paper could provide some help for the application of fish skin collagen and the identification of key peptides from protein hydrolysates.

## Figures and Tables

**Table 1:** Molecular weight distributions of three stages of hydrolysates.

Hydrolysates	ASCH		SGI-1		SGI-2	
Num	MW (Da)	Content (%) *	MW (Da)	Content (%)	MW (Da)	Content (%)
1	3198.76	66.82	1552.34	74.66	1026.26	59.49
2	2245.49	16.45	976.83	18.04	640.53	18.34
3	647.39	8.09	505.59	5.08	284.97	16.60
4	199.63	8.08	180.09	2.15	96.58	4.56

* the percentage of the peak area.

**Table 2:** Amino acid compositions of three stages of hydrolysates (No. of residues per 1000 residues).

Amino Acids	ASCH	SGI-1	SGI-2
Asp	54.19	56.37	56.81
Thr	25.56	26.43	27.37
Ser	64.78	64.81	62.17
Glu	75.24	76.53	76.38
Gly	314.42	325.46	319.66
Ala	109.31	107.95	105.06
Cys	31.80	19.17	23.18
Val	17.90	19.44	20.56
Met	15.01	14.53	14.39
Ile	11.82	12.49	13.42
Leu	22.25	22.71	23.58
Tyr	3.40	3.77	4.90
Phe	13.04	12.81	13.89
Lys	27.95	28.70	30.59
NH_3_	57.51	54.29	69.71
His	8.83	8.87	9.17
Arg	53.01	52.55	35.25
Pro	93.99	93.13	93.90
THAA*	315.11	302.22	307.98
Total	1000	1000	1000

* THAA: total hydrophobic amino acid.
